# Regulatory B Cells and Their Cytokine Profile in HCV-Related Hepatocellular Carcinoma: Association with Regulatory T Cells and Disease Progression

**DOI:** 10.3390/vaccines8030380

**Published:** 2020-07-11

**Authors:** Helal F. Hetta, Mohamed A. Mekky, Asmaa M. Zahran, Mohamed O. Abdel-Malek, Haidi K. Ramadan, Engy A. Shafik, Wael A. Abbas, Muhammad Abbas El-Masry, Nahed A. Mohamed, Amira A. Kamel, Najat Marraiki, Amany Magdy Beshbishy, Gaber El-Saber Batiha, Heba A. Osman, Gopala Koneru, Mohamed A. El-Mokhtar

**Affiliations:** 1 Department of Internal Medicine, University of Cincinnati College of Medicine, Cincinnati, OH 45267-0595, USA; gkoneru1@gmail.com; 2 Department of Medical Microbiology and Immunology, Faculty of Medicine, Assiut University, Assiut 71515, Egypt; 3 Department of Tropical Medicine and Gastroenterology, Faculty of Medicine, Assiut University, Assiut 71515, Egypt; Mmekky75@yahoo.com (M.A.M.); momar7619@gmail.com (M.O.A.-M.); haidikaram@aun.edu.eg (H.K.R.); 4 Department of Clinical Pathology, South Egypt Cancer Institute, Assiut University, Assiut 71515, Egypt; asmaam.zahran@yahoo.com (A.M.Z.); engyadelshafik@yahoo.com (E.A.S.); 5 Department of Internal Medicine, Gastroenterology Division, Faculty of Medicine, Assiut University, Assiut 71515, Egypt; drwaelabbas@yahoo.com (W.A.A.); Masaeed2@yahoo.com (M.A.E.-M.); 6 Department of Medical Biochemistry, Faculty of Medicine, Assiut University, Assiut 71515, Egypt; nahed_eltamawy@yahoo.com (N.A.M.); amira_kamel222@yahoo.com (A.A.K.); 7 Department of Botany and Microbiology, College of Science, King Saud University, Riyadh 11451, Saudi Arabia; Najat@ksu.edu.sa; 8 National Research Center for Protozoan Diseases, Obihiro University of Agriculture and Veterinary Medicine, Nishi 2-13, Inada-cho, Obihiro 080-8555, Hokkaido, Japan; amanimagdi2008@gmail.com; 9 Department of Pharmacology and Therapeutics, Faculty of Veterinary Medicines, Damanhour University, Damanhur 22511, Egypt; gaberbatiha@gmail.com; 10 Tropical Medicine and Gastroenterology Department, Hepatology Division, Faculty of Medicine, South Valley University, Qena 83523, Egypt; hebaahmed198098@yahoo.com

**Keywords:** Breg, Treg, HCV, HCC

## Abstract

Although regulatory B cells (Bregs) have been proven to play a suppressive role in autoimmune diseases, infections and different tumors, little is known regarding hepatocellular carcinoma (HCC), especially in hepatitis C-related settings. Herein, we analyzed the frequency of circulating Bregs, serum levels of IL-10, IL-35 and B-cell activating factor (BAFF) and investigated their association with regulatory T cells (Tregs) and disease progression in HCV-related HCC. For comparative purposes, four groups were enrolled; chronic HCV (CHC group, n = 35), HCV-related liver cirrhosis (HCV-LC group, n = 35), HCV-related HCC (HCV-HCC group, n = 60) and an apparently healthy control (Control-group, n = 20). HCC diagnosis and staging were in concordance with the Barcelona Clinic Liver Cancer (BCLC) staging system. Analysis of the percentage of Breg cells and peripheral lymphocyte subsets (Treg) was performed by flow cytometry. Serum cytokine levels of IL-10, IL-35 and B-cell activating factor (BAFF) were measured by ELISA. The frequency of Bregs was significantly higher in the HCV-HCC group compared to the other groups and controls. A significant increase was noted in late-HCC versus those in the early stages. The frequency of Bregs was positively correlated with Tregs, serum IL-10, IL-35 and BAFF. In conclusion, Peripheral Bregs were positively correlated with the frequency of Tregs, IL-10, IL-35 and BAFF, and may be associated with HCV-related HCC progression.

## 1. Introduction

Hepatocellular carcinoma (HCC) the fourth most common cause of cancer-related death worldwide; >80% of HCC cases occur in low-resource and middle-resource countries, particularly in Eastern Asia and sub-Saharan Africa, where medical and social care resources are often constrained [1]. One of the common risk factors for HCC is chronic hepatitis B and chronic hepatitis C (CHC) infection [2,3,4]. In areas with a high endemicity of HCV, like in Egypt, a rising trend of HCC will be expected [5,6,7]. 

There is a balance between immune effector cells and immunosuppressive cells to regulate the tumor microenvironment. It has been reported that there is a significant contribution of immune regulatory cells, including regulatory T cells (Tregs), to tumor progression [8,9,10]. Recently, there is evidence that B cells play a role in modulating the immune response to tumors and lymphoid malignancies [11].

Regulatory B cells (Bregs) are subset of B cells that are reported to play a crucial role in regulating the immune responses in cases of inflammation, autoimmunity and cancer [12]. Bregs exert a suppressive potential towards many cells, including T cells, via secreting anti-inflammatory mediators, such as IL-10, and also potentiate the conversion of T cells into Tregs with subsequent attenuation of the anti-tumor immune responses [13]. 

The phenotypic diversity of the cell surface markers that are unique to Bregs remains unclear. Bregs arise from the transitional 2 marginal zone precursor (T2-MZP) B cells, which have most of the characteristic markers for Bregs. Human Bregs may be also termed human IL-10 producing B cells. (B10) is a subset of B cells is enriched in the CD19^+^CD24^high^CD27^−^ CD38^high^CD1d^high^CD5^+^ transitional B cell subset. Tumor-Evoked Regulatory B Cells (tBreg) exert antitumor activity by promoting conversion of the resting CD4^+^ T cell into FoxP3^+^ Treg by secretion of TGF-β then the Treg inhibit T cell proliferation and promote tumor metastasis by suppression of the anti-tumor effects of CD8^+^ T cells and NK cells [11,14].

Both cell-mediated and humoral immune responses are key players in the immunopathology of HCC [15,16]. Recently, Bregs were found to be abundant in the tumor microenvironment and were a leading cause of progression of various cancers, including HCC [11].

Bregs may suppress the antitumor immunity and promote HCC progression via several mechanisms including the CD40/CD40L signaling-mediated cytokine production of IL10, TGF-β which downregulates TNF- α, PD-1^hi^ B-cell, Granzyme B-secreting B cells (GrB+ B cells), Treg upregulation, TH17 downregulation, and IL-35 which triggers the genesis of Tregs from naive T cells with the subsequent suppression of the anticancer immune response. The hallmark of Breg function is IL-10, which inhibits proinflammatory cytokines and supports Treg differentiation [13].

The immune-related inter-players between the CHC and the subsequent HCC-tumorigenesis were investigated thoroughly in various immunologic studies [17,18]. One of these players is the Breg cells, which have been proven to downregulate inflammatory immune responses and induce a tolerance through the array of cytokines, e.g., IL-10 and/or TGF-β, and counteract the pathogenic T cells to suppress the harmful immune effects [19,20,21]. Furthermore, the production of B cell-activating factor (BAFF), a member of TNF family cytokines, is documented in the regulation of B cell maturation and survival, was noted to be increased in post-hepatitis liver damage [22,23].

Egyptian studies in the context of HCC tumorigenesis in post CHC infection are still scarce. Therefore, we aimed to analyze the frequency of Breg cells among HCV-related HCC patients and studying its association with Treg cells, IL-10, IL-35, BAFF, and tumor progression.

## 2. Patients and Methods 

The study was approved by our Local Ethics Committee (IRB No 17300237) of the Faculty of Medicine, Assiut University, Egypt, and in concordance with the Helsinki II declaration. An informed written consent was taken from all cases and controls.

Between January 2019 and December 2019, a single center cross-sectional study at Al-Rajhi Liver hospital, Assiut University Hospital, Egypt, was designed to enroll three groups of patients: patients with chronic HCV (CHC group, n = 35), patients with HCV-related liver cirrhosis (HCV-LC group, n = 35), and patients with HCV-related HCC (HCV-HCC group, n = 60). Another apparently healthy control group was added (control group, n = 20) from the blood donation banking.

### 2.1. Study Definitions and Patients‘ Selection

- CHC was defined when HCV-Ab and HCV-RNA-PCR was positive for more than 6 months. HCV viral load was determined by using Artus1 HCV-RG RT-PCR Kit (cat#4518265, QIAGEN, Germany) according to the manufacturer’s protocol. QRT-PCR was performed on 7500 Fast real-time PCR Thermal cycler (Applied Biosystems, CA, USA).
- HCV-related Liver cirrhosis (HCV-related LC) was diagnosed based on the results of clinical evaluation, laboratory tests (alanine aminotransferase (ALT), aspartate aminotransferase (AST), alkaline phosphatase (ALP), bilirubin, albumin, and estimation of prothrombin time), and imaging (using well-defined criteria of liver cirrhosis). Severity of liver cirrhosis was assessed by the Child–Pugh score (CPS) [24].
- HCC diagnosis was also based on both laboratory testing for alpha-fetoprotein (AFP; using Access 2 tumor marker analyzer, Beckman Coulter, USA S.N.: 510552), and imaging (using CT-scan and/or MRI study). Staging of HCC was in concordance with the Barcelona Clinic Liver Cancer (BCLC) staging system [25,26]. HCC-patients were classified according to the Barcelona Clinic Liver Cancer (BCLC) staging system into A, B, and C classes. Early stage (A) includes patients with asymptomatic early tumors suitable for radical therapies-resection, transplantation or percutaneous treatments. Intermediate stage (B) comprises patients with asymptomatic multinodular HCC. Advanced stage (C) includes patients with symptomatic tumors and/or an invasive tumoral pattern [27].
- *Exclusion criteria:* Patients co-infected with hepatitis B virus, with other co-morbid extrahepatic neoplasms, or patients with chronic autoimmune diseases were excluded from the study.

### 2.2. Evaluation of the Freuency of Bregs by Flow Cytometry

**Figure 1:**
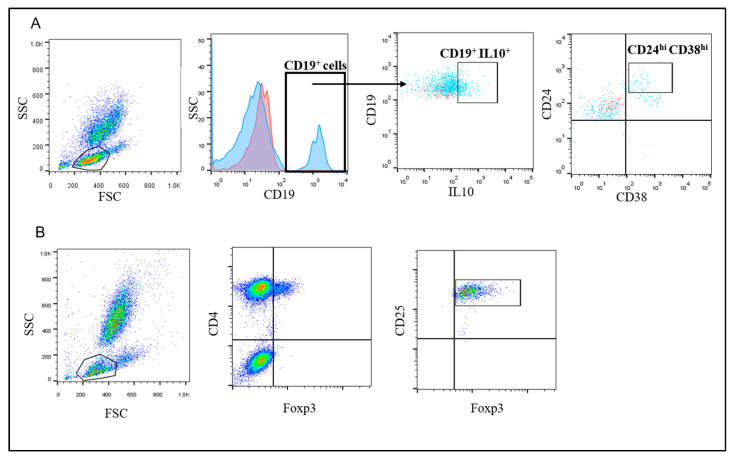
Representative flow cytometry gating strategy to detect Tregs and Bregs. (**A**) FSC and SSC were used to define the lymphocyte population. CD19^+^ cells were gated (the blue histogram represents cells stained with anti-CD19 Ab and the red curve represent cells stained with isotype-matched control) and IL-10^+^ cells were defined. Then cells that are CD24^+^CD38^high^ were assessed (Breg cells were defined as CD19^+^IL-10^+^CD24^+hi^CD38^+hi^ cells. For the proper gating of the IL-10^+^ and CD24^+hi^CD38^+hi^ cells (shown as light blue in the dot blots), fluorescence minus one controls were employed (shown in red color in the dot blots) (**B**) FSC and SSC were used to define the lymphocytes population followed by gating the CD4^+^Foxp3+ cells. Then, the CD25^+^ cells were determined (T regs were defined as CD4^+^FoxP3^+^CD25^+high^ cells).

Using flow cytometry, circulating Bregs were detected using FITC-conjugated-CD38, PE-conjugated-CD24 (Bioscience, USA), and PerCP-conjugated CD19 (BD Bioscience, USA). Briefly, 100 µl of blood sample was incubated with 10 µL of CD24, CD38 and CD19 for 20 min at 4 °C in the dark. Following incubation, RBCs were lysed and washed. Cells were fixed and permeabilized then stained with APC-conjugated IL-10 (BD Bioscience, San Jose, CA, USA) and analysis by FACS Calibur flow cytometry with CellQuest software (Becton Dickinson Biosciences, San Jose, CA, USA). An isotype-matched negative control was used for each sample. Forward and side scatter histograms were used to define the lymphocytes population. CD19^+^ IL-10^+^ B cells were gated, then the expression of CD38 and CD24 on the CD19^+^B cells were detected. Bregs were identified as CD19^+^ IL-10^+^CD24^+hi^CD38^+hi^ cells. CD19^+^ B cells were selected based on the use of the isotype-matched negative control. However, for proper gating of the IL-10^+^ and CD24^+hi^CD38^+hi^ cells, fluorescence minus one controls were employed, shown in light blue and red colors in the dot blots for the gated cells and controls, respectively (Figure 1A).

### 2.3. Evaluation of the Frequency of Tregs by Flow Cytometry

Tregs were enumerated using FITC-conjugated Foxp3 (eBioscience, San Diego, CA, USA), PE-conjugated CD25 (IQ Product, The Netherland), and Per-CP-conjugated CD4 (BD Biosciences, CA, USA). Briefly, 100 µL of blood sample was incubated with 10 µL of CD4, CD25 for 15 min at 4 °C in the dark. Following incubation, RBCs were lysed by addition of FACS lysing solution (BD Biosciences, CA, USA), washed with PBS, fixed and permeabilized using the Cytofix/Cytoperm Kit (BD Biosciences, CA, USA) and then strained intracellularly with FITC-conjugated Foxp3 antibodies. Flow cytometric analysis was performed by FACSCalibur flow cytometry and FlowJo software 7.6.1 (Tree Star Inc., Ashland, OR, USA). Anti-human IgG was used as an isotype-matched negative control for each sample. Forward and side scatters were used to define the lymphocyte population. Then CD4^+^CD25^+hi^ Foxp3^+^ Tregs were evaluated (Figure 1B). 

### 2.4. Serum Cytokine Measurements

IL-10 serum levels were measured using ELISA kit (Raybiotech, Norcross, GA, USA) according to the manufacturer’s instructions. All samples were measured in triplicate. These kits had a sensitivity of >1 pg/mL against IL-10. The BAFF serum levels were measured by ELISA (R&D Systems, Minneapolis, MN, USA) following the manufacturer instructions. The sensitivity for BAFF serum levels was 62.5–4000 pg/mL. IL-35 serum concentration was measured for all participants using ELISA kit (Glory Science CO., Ltd, TX, USA, Cat No #:99569) according to the manufacturer’s protocol. The detection limit for IL-35 was 15 pg/mL.

### 2.5. Statistical Analysis

Statistical analyses were performed with GraphPad Prism version 7.0 b software (Graph Pad Software Inc., San Diego, CA, USA). Qualitative data are expressed as frequency and percentage, while quantitative data are expressed by mean ± standard error (SEM). A Mann–Whitney analysis was used to detect the statistical significance between groups. Spearman’s correlation was used to correlate the studied parameters. A *p* value < 0.05 was considered significant. 

## 3. Results

### 3.1. Demographic Data

Demographic and biochemical measurements of the enrolled groups are summarized in Table 1. There was no significant difference in age and gender between the enrolled groups. The HCV-HCC group was stratified according to the BCLC staging system as follows: 30 (50%) patients were stage A, 20 (33%) patients were stage B and 10 (17%) patients were stage C. The HCV- LC group were divided according to Child–Pugh score as follows: 15 (43%) patients were class A, 13 (37%) class B, and 7 (20%) class C. 

### 3.2. Frequency of Bregs and Tregs c among the Studied Groups 

As shown in Table 2, the frequency of circulating Bregs was significantly increased among HCV-HCC patients compared to CHC (*p* = 0.01) and healthy controls (*p* < 0.0001), with a significantly higher frequency among HCV-HCC patients with stage C (8.03 ± 4.3) than stage A (4.75 ± 2.3) and stage B (5.9 ± 2.6), (*p* = 0.04). 

On the other hand, circulating Tregs were significantly increased among HCV-HCC patients compared to CHC (*p* = 0.0002), LC (*p* = 0.02) and control (*p* < 0.0001). 

### 3.3. IL-10, IL-35 and BAFF Serum Levels

Table 2 summarizes serum cytokine levels among patient groups. HCV-HCC patients had significantly higher levels of IL-10, IL-35 and BAFF compared to the CHC group; (*p* < 0.0001), (*p* = 0.04) and (*p* < 0.0001), respectively. Moreover, the HCV-HCC group showed significantly higher levels of IL-10, IL-35 and BAFF compared to HCV-LC; (*p* < 0.0001), (*p* = 0.03) and (*p* = 0.01), respectively. Additionally, the HCV-HCC group showed significantly (*p* < 0.0001) elevated levels of IL-10, IL-35 and BAFF compared to controls. 

### 3.4. Correlation of the Frequency of Bregs with the Frequency of Tregs, IL-10, IL-35 and BAF Serum Levels among HCV-HCC Patients 

The frequency of Bregs was positively correlated with the frequency of Tregs (r = 0.26, *p* = 0.03). Moreover, the frequency of circulating Bregs was positively correlated with serum levels of IL-10, IL-35 and BAFF among HCV-HCC group (r = 0.3, *p* = 0.01), (r = 0.27, *p* = 0.03) and (r =0.4, *p* = 0.001), respectively. The frequency of Bregs was positively correlated with AFP (r = 0.20, *p* = 0.03) and ALT (r = 0.3, *p* = 0.01).

## 4. Discussion

Egypt is known to have a high prevalence of HCV infection, and hence the post-hepatitis HCC occurrence is highly expected [28,29,30,31]. This may need intense research, especially in the area of HCC oncogenesis, by exploring the immune-pathogenesis and potential triggers [9,32,33,34]. Many theories have tried to postulate the potential role of immune cells, especially that of immune-regulatory cells, such as IL-10^+^ Bregs and Tregs, that have been proven to play a pivotal role in immune down-regulation [35,36]. Data available about their exact role in hepatocarcinogenesis are still limited, especially in post HCV settings. 

In our study we found that HCV-HCC patients had higher frequencies of peripheral Bregs and Tregs compared to CHC, HCV-LC and healthy controls. It is possible that the systemic inflammatory state caused by hepatitis induces the expansion of peripheral Bregs; beside that, HCC itself may produce abundant specific cytokines and chemoattractant including IL-8 [37] and CCL 20 [38], some of which are responsible for the “homecoming” signals which orient regulatory lymphocytes into the tumor. Furthermore, it is possible that Bregs and Tregs collaborate and help each other, contributing to the development of HCC.

Moreover, Tregs can inhibit the specific anti-tumor immune response in the tumor microenvironment via (1) the release of granzyme B and perforins to cause the direct lysis of effector T-cells through; (2) the induction of apoptosis of effector T cells through the deprivation of IL-2 by high-affinity CD25; (3) the modulation of maturation and function of dendritic cells by cell–cell contact-dependent mechanisms involving cytotoxic T lymphocyte antigen-4; and (4) the release of inhibitory cytokines including IL-10, TGF-β, IL-35 and prostaglandin E2 to modulate effector cell immune responses [39]. Tregs modulate their suppressive activity by the high expression of transcription factor Forkhead box (Fox)p3. Moreover, the deletion of Tregs is considered a potential immunotherapy strategy for HCC [40]. Our results regarding elevated Treg frequency in CHC infection came in concordance with previous studies which reported an increased percentage of CD4^+^ CD25^high^ T cells in CHC patients which was correlated with higher viremia levels [41,42,43]. Contrary to our observations, patients with active autoimmune hepatitis showed lower levels of Tregs and expressed lower FOXP3 levels, and functional analysis revealed a lower ability to inhibit target cell proliferation. However, the mechanism of liver damage in autoimmune hepatitis is different from HCV-associated HCC, and involves the recognition of autoantigenic epitopes on liver cells [44].

Several studies have shed light on the role of Tregs in cancer; however, little is known about the role of Bregs in HCC and the disease progression. Bregs are characterized by important regulatory roles which are mainly attributed to the production of regulatory cytokines such as IL-10 and TGF-β1 that exhibit inhibitory functions. These cytokines can decrease the expression of TH1 and TH2 cytokines and inhibit the cytotoxic activity of TH1/CD8^+^ T cells. In addition, Breg could exert their regulatory functions through the production of antibodies which promote immune complex production and stimulating signals that promote tumor growth and progression [14,45]. It was reported that CD19^+^CD24^hi^CD38^hi^ Bregs were enriched in the tumor microenvironment and associated with the progression of several tumors including HCC, providing an additional support to our results [46].

Interestingly, we found a positive correlation between the frequency of Bregs and Tregs. It was reported previously that Bregs can facilitate the earlier stage of the recruitment of Tregs in autoimmune disorders [44,47]. When co-cultured with CD4^+^ T cells, Bregs supported the maintenance of CD4^+^ Foxp3^+^ Tregs in vitro. The transfer of these B cells into CD19^−/−^ mice results in a significant increase in the numbers of CD4^+^Foxp3^+^ Tregs in the thymus, spleen, and lymph nodes [48]. Several studies support our findings that the interplay between Bregs and Tregs could result in the acceleration of HCC progression [14,49,50]. Recent reports found that B cells can convert CD4^+^CD25^−^ T cells into Tregs [49] and the frequencies of IL-10^+^ B cells are also positively correlated with the frequencies of CD4^+^Foxp3^+^ Tregs [51]. Moreover, Bregs can enhance the expression of Foxp3 and CTLA-4 (markers for the suppressive power of Tregs) in Tregs through cell-to-cell contact [52].On the other hand, CD4^+^CD25^+^ Tregs can also induce the expansion of B10 cells which facilitate the interplay between two cells [53].

We found that the frequency of Bregs was positively correlated with ALT and AFP, which are increased in cases with severe liver damage. Moreover, AFP levels increase in HCC generation so that it remains the most commonly used screening biomarker for HCC [54].

Furthermore, in this study, the percentage of Breg cells was significantly correlated with serum levels of IL-10 and IL-35 among HCC patients. Interestingly, some studies provided direct evidence that IL-35 can induce the conversion of conventional B cells or IL-10-Bregs into the novel IL-35-producing B cells, named i35-Breg [55]. Furthermore, IL-35 is specifically produced by Treg cells and required for its maximal suppressive activity [56]. There is a compelling evidence that mouse IL-35 can convert conventional T cells into the IL-35-producing Treg population now known as iTR35. Collectively, our data support the notion that IL-10 and IL-35 are autocrine factors for the development of IL-10^+^ Bregs [57].

In our study, we reported an increase in the BAFF serum levels among HCV-HCC patients compared to other groups. Previously it was reported that CHC patients had elevated serum levels of BAFF than controls. BAFF is expressed locally in myeloid cells including macrophages and dendritic cells following activation by cytokines, such as IFNs and interleukin IL-10 [58]. Besides that, accumulating evidence regarding the importance of B cell-activating factor (BAFF), a member of TNF family cytokines, in the regulation of B cell maturation and survival. Analyses of BAFF-deficient mice reveal a fundamental role of BAFF during the transition from immature T1 to T2 B cells [59]. Moreover, BAFF has a crucial role for MZ B cell development [60]. New evidence from BAFF transgenic mice indicates that BAFF induces CD4^+^Foxp3^+^T cells to suppress T cell responses through an indirect B cell dependent manner, suggesting a regulatory role of BAFF in vivo [61].

Remarkably, it is well-known that TGF-β and IL-10 play a major role in chronic inflammation and liver fibrosis. Moreover, epigenetic components that permit access of the β-catenin complex to the promoter and enhancer regions of its target genes are downstream regulators of β-catenin-dependent transcription, thus impacting HCC sorafenib sensitivity [62,63]. In the frame of this thinking, the few therapeutic strategies for advance HCC on poor knowledge of its biology. For several years, sorafenib—a tyrosine kinase inhibitor (TKI) and BRAF inhibitor (BRAFi)—has been the approved treatment option for advanced HCC patients. Its activity is the inhibition of the retrovirus-associated DNA sequences protein (RAS)/Rapidly Accelerated Fibrosarcoma protein (RAF)/mitogen-activated and extracellular-signal-regulated kinase (MEK)/extracellular-signal-regulated kinases (ERK) signaling pathway. Strikingly, there is growing evidence that MAPK pathway activation impairs antitumor immunity and that targeting this pathway may enhance responses to immunotherapies, potentially reflecting a decrease in immune cell effector function. Therefore, from a clinical standpoint, several cancers with terribly poor prognosis could benefit from novel insights derived from these data and combining immune-depletion with BRAF inhibitors [64,65].

A total of 70–80% of HCC patients are diagnosed at an advanced stage with a dismal prognosis. Sorafenib was the standard care until 2018 with the arrival of an alternative first-line agent; namely lenvatinib. Cabozantinib, regorafenib, and ramucirumab were approved by FDA and they also displayed promising results. Additionally, nivolumab and pembrolizumab, two therapeutics against the Programmed death (PD)-ligand 1 (PD-L1)/PD1 axis have been recently approved. The response rate of single agent targeting PD-L1/PD1 axis is low. Therefore, many combinatory approaches are under investigation, including the combination of different immune checkpoint inhibitors (ICIs) [66].

## 5. Conclusions

Peripheral Bregs were found to be correlated with Tregs, IL-10, IL-35, BAFF and HCV-related hepatocarcinogenesis, and this may serve as a potential site for the advent of future molecular therapeutic options.

## Figures and Tables

**Table 1:** Clinical and biochemical characterization of all groups enrolled in the study.

	CHC (n = 35)	HCV-LC (n = 35)	HCV–HCC (n = 60)	Healthy Controls (n = 20)	*p*-Value
**Age (range, years)**	45–65	50–65	50–70	40–60	0.4
**Sex (male/female)**	25/10	20/15	40/20	14/6	0.2
**Hb (g/dL)**	11.37 ± 2.5	10 ± 1.57	12.68 ± 1.55	13.7 ± 2.1	0.41
**Total bilirubin(mg/dL) [Normal range: 0.1 to 1.2]**	1.43 ± 0.92	1.66 ± 1.33	1.62 ± 1.67	0.77 ± 0.08	<0.001
**Albumin(g/dL) [Normal range: 3.4 to 5.4]**	3.15 ± 1.42	3.56 ± 1.22	3.04 ± 0.7	4.08 ± 0.41	<0.001
**ALT [Normal: up to 40 IU/L]**	48.6 ± 42	51.2 ± 47	71.1 ± 12	22.1 ± 5.6	<0.001
**AST (IU/L) [Normal: up to 40 IU/L]**	71.4 ± 58	76.4 ± 58	81.4 ± 75	21.2 ± 3.9	<0.001
**AFP (IU/L) [Normal range <10]**	3.5 ± 1.2	8.45 ± 7.43	785.3 ± 203.3	1.1 ± 0.3	<0.001
**Prothrombin concentration (%)**	85.4% ± 3.01	56.8% ± 18.2	72.53% ± 17.5	90.6 ± 5.2	<0.00001
**INR**	1.05 ± 0.051	1.45 ± 0.33	1.2 ± 0.177	1.15 ± 0.03	<0.00001
**HCV-RNA copy number/mL**	1.3 × 10^6^ ± 2 × 10^6^	2.7 × 10^6^ ± 2 × 10^6^	7 × 10^6^ ± 16 × 10^6^	NA	NA
**Child–Pugh class**	NA	Class A (n = 15)	Class A (n = 30)	NA	NA
		Class B (n = 13)	Class B (n = 20)		
		Class C (n = 7)	Class C (n = 10)		
**Hepatic focal lesions**	NA	NA	single < 5 cm (n = 30)	NA	NA
			single > 5 cm (n = 18)		
			multiple (n = 12)		
**BCLC staging**	NA	NA		NA	NA
**Stage-A**			20		
**Stage-B**			15		
**Stage-C**			25		

Kruskal–Wallis test, Data represented as means ± SEM. *p* ≤ 0.05 is significant. ALT; alanine amino transferase, AST; aspartate aminotransferase, AFP; α-fetoprotein, NA; not applied, BCLC Barcelona clinic liver cancer, INR: International normalized ratio.

**Table 2:** Bregs, Tregs, IL-10, IL-35 and BAFF levels in the studied groups.

Variable (Mean ± SD)	CHC (n = 35)	HCV-LC (n = 35)	HCV-HCC (n = 60)	Healthy Controls (n = 20)	*P 1 V*alue	*P 2 V*alue	*P 3 V*alue
**%CD 19^+^B cell**	10.90 ± 1.78	11.56 ± 1.71	11.75 ± 1.81	12.11 ± 1.79	0.03	ns	ns
**%Bregs**	5.13 ± 2.01	5.62 ± 1.90	6.26 ± 2.71	3.71 ± 0.9	0.01	ns	<0.0001
**%Tregs** (CD4^+^CD25^+high^ FoxP3^+^cells)	1.67 ± 0.35	1.89 ± 0.37	2.04 ± 0.34	1.51 ± 0.29	0.0002	0.02	<0.0001
**IL-10 pg/mL**	4 ± 1.83	6.27 ± 2	10.25 ± 3.69	3.72 ± 2.43	<0.0001	<0.0001	<0.0001
**IL-35 pg/mL**	108.9 ± 43. 7	136.4 ± 73.2	154.6 ± 87.5	65.6 ± 45.4	0.04	0.03	<0.0001
**BAFF pg/mL**	348.5 ± 292.1	451.7 ± 379	772 ± 463.6	272 ± 267.2	<0.0001	0.001	<0.0001

ns: non-significant. BAFF; B-cell activating factor, P1–comparison between CHC and HCV-HCC, P2–comparison between HCV-LC and HCV-HCC, P3–comparison between HCV-HCC and Healthy controls, Mann–Whitney Test, Data represented as means ± SEM. *p* ≤ 0.05 is significant.
